# Impaired SARS-CoV-2 vaccine responsiveness is not associated with subclinical atherosclerosis or cardiovascular disease

**DOI:** 10.1093/ehjopen/oeaf167

**Published:** 2025-12-09

**Authors:** Samuel H A Andersson, Anthi Chalou, Megan Mulholland, Pernilla Katra, Irena Ljungcrantz, Linda Andersson, Gunnar Engström, Jan Nilsson, Alexandru Schiopu, Harry Björkbacka, Daniel Engelbertsen

**Affiliations:** Department of Clinical Sciences, Malmö, Lund University, Jan Waldenströms Gata 35, CRC 91:12, Box 50 332, Malmö, 202 13, Sweden; Department of Clinical Sciences, Malmö, Lund University, Jan Waldenströms Gata 35, CRC 91:12, Box 50 332, Malmö, 202 13, Sweden; Department of Clinical Sciences, Malmö, Lund University, Jan Waldenströms Gata 35, CRC 91:12, Box 50 332, Malmö, 202 13, Sweden; Department of Clinical Sciences, Malmö, Lund University, Jan Waldenströms Gata 35, CRC 91:12, Box 50 332, Malmö, 202 13, Sweden; Department of Clinical Sciences, Malmö, Lund University, Jan Waldenströms Gata 35, CRC 91:12, Box 50 332, Malmö, 202 13, Sweden; Department of Clinical Sciences, Malmö, Lund University, Jan Waldenströms Gata 35, CRC 91:12, Box 50 332, Malmö, 202 13, Sweden; Department of Clinical Sciences, Malmö, Lund University, Jan Waldenströms Gata 35, CRC 91:12, Box 50 332, Malmö, 202 13, Sweden; Department of Clinical Sciences, Malmö, Lund University, Jan Waldenströms Gata 35, CRC 91:12, Box 50 332, Malmö, 202 13, Sweden; Department of Translational Medicine, Cardiac Inflammation Research, Lund University, Jan Waldenströms Gata 35, CRC 60:13, Box 50 332, Malmö, 202 13, Sweden; Department of Internal Medicine, Skåne University Hospital, Entrégatan 7, Lund, 221 85, Sweden; Institute of Cellular Biology and Pathology ‘N. Simionescu’, Bucharest, No. 8, B.P. Hasdeu Street, 050568, Romania; Department of Clinical Sciences, Malmö, Lund University, Jan Waldenströms Gata 35, CRC 91:12, Box 50 332, Malmö, 202 13, Sweden; Department of Clinical Sciences, Malmö, Lund University, Jan Waldenströms Gata 35, CRC 91:12, Box 50 332, Malmö, 202 13, Sweden

**Keywords:** Atherosclerosis, Immunity, Vaccination

## Abstract

**Aims:**

Although age-related immune deterioration has been implicated as a mechanistic contributor to cardiovascular disease (CVD), evidence for an impairment of adaptive immune function in individuals with clinically verified presence of atherosclerosis is lacking.

**Methods and results:**

To test the association between atherosclerosis and immune function, we evaluated SARS-CoV-2 vaccine responsiveness in 65- to 71-year-old individuals (*n* = 644) derived from a population-based cohort, characterized for subclinical atherosclerosis by coronary computed tomography angiography and carotid ultrasound. Vaccine-specific T cells were quantified by activation-induced marker assays and antibody responses by ELISA. We did not find any significant associations between the degree of subclinical atherosclerosis or history of cardiovascular disease and vaccine-specific IgG or T cells. Vaccine immunity was not associated with lipid levels but was inversely correlated with several plasma cytokines.

**Conclusions:**

Our study demonstrates that subclinical atherosclerosis or prevalent CVD is not associated with impaired responsiveness to vaccination.

## Graphical Abstract

**Graphical Abstract:**
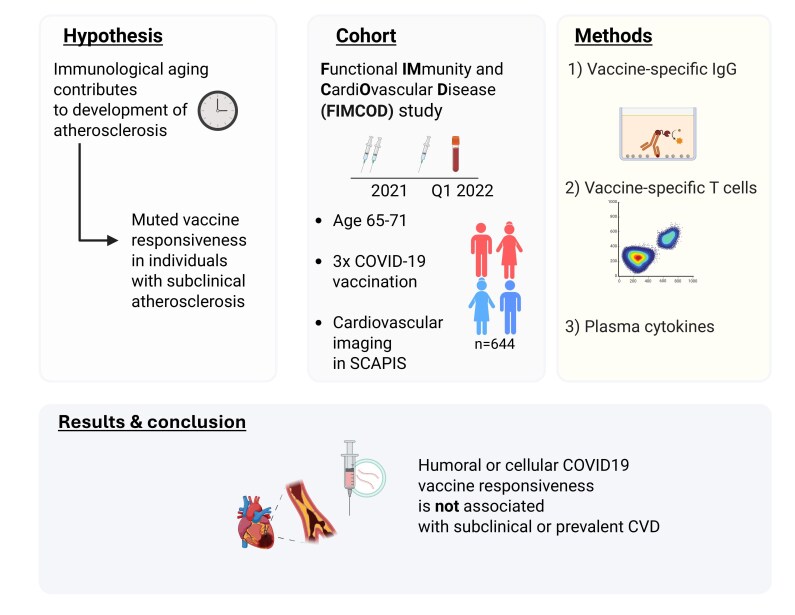

## Introduction

Cellular and transcriptional biomarkers indicating immunological aging of adaptive immunity have been linked to atherosclerosis.^1–4^ Although transcriptional or phenotypic changes in cell populations may be indicative of immunological aging, the potential relationship between functional impairment of adaptive immunity and cardiovascular disease (CVD) has not previously been evaluated. In this study, we tested whether impaired humoral or cellular SARS-CoV-2 vaccine responsiveness, potentially reflecting premature immune aging, was associated with subclinical or prevalent CVD burden in a population-based cohort of individuals aged 65–71 years.

## Methods

### Study population

**Figure 1:**
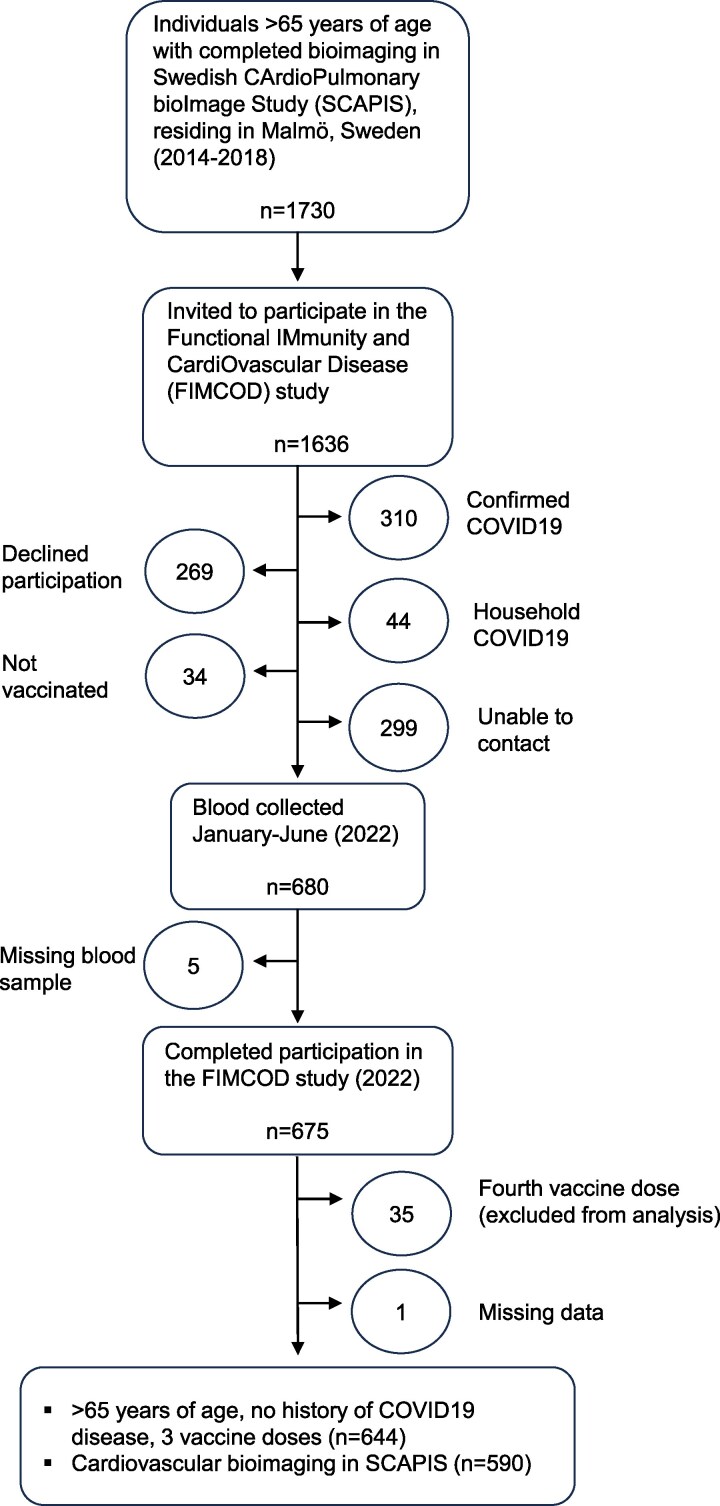
Functional IMmunity and CardiOvascular Disease (FIMCOD) study timeline and study participant selection.

In the Functional IMmunity and CardiOvascular Disease (FIMCOD) study, we collected peripheral blood mononuclear cells (PBMCs) and plasma from study participants that had (i) received three doses of any SARS-CoV-2 vaccine and (ii) been characterized for subclinical atherosclerosis by coronary computed tomography angiography and carotid ultrasound in the Swedish CArdioPulmonary bioImage study^5^ (SCAPIS) (*Figure 1*). Study participants answered a health questionnaire containing questions related to SARS-CoV-2 vaccination and disease history. Individuals with a confirmed COVID-19 diagnosis or household SARS-CoV-2 infection were excluded. A history of CVD was defined as self-reported prevalent myocardial infarction, angina, heart failure, coronary bypass surgery or balloon angioplasty, treatment of narrowing carotid or leg arteries, or stroke (ischaemic and haemorrhagic).

### Vaccine responsiveness and immunophenotyping

IgG against the SARS-CoV-2 S1 protein was measured by ELISA (Quantivac, EUROIMMUN). Vaccine-specific T cells were identified by upregulation of activation-induced markers (AIMs) in response to antigenic stimulus.^6^ PBMCs (4–6 × 10^5^ per condition) were thawed and cultured for 24 h with or without SARS-CoV-2 spike peptide pools (PepTivator®SARS-CoV-2, Miltenyi) in complete media containing anti-CD40 (Miltenyi) and anti-CD28/CD49d (BD Bioscience). Percent vaccine-specific (AIM^+^) T cells were calculated by measuring the percentages of AIM^+^ T cells after stimulation with peptide pools of SARS-CoV-2 S-protein and subtracting the background levels of AIM^+^ T cells in the unstimulated condition. Plasma cytokines and soluble proteins were quantified using the LEGENDplex platform (Biolegend).

### Statistics

Statistical analyses were performed using SPSS Statistics 27 (IBM, Armonk, USA) and *P*-values of ≤0.05 were considered significant.

## Results

**Figure 2:**
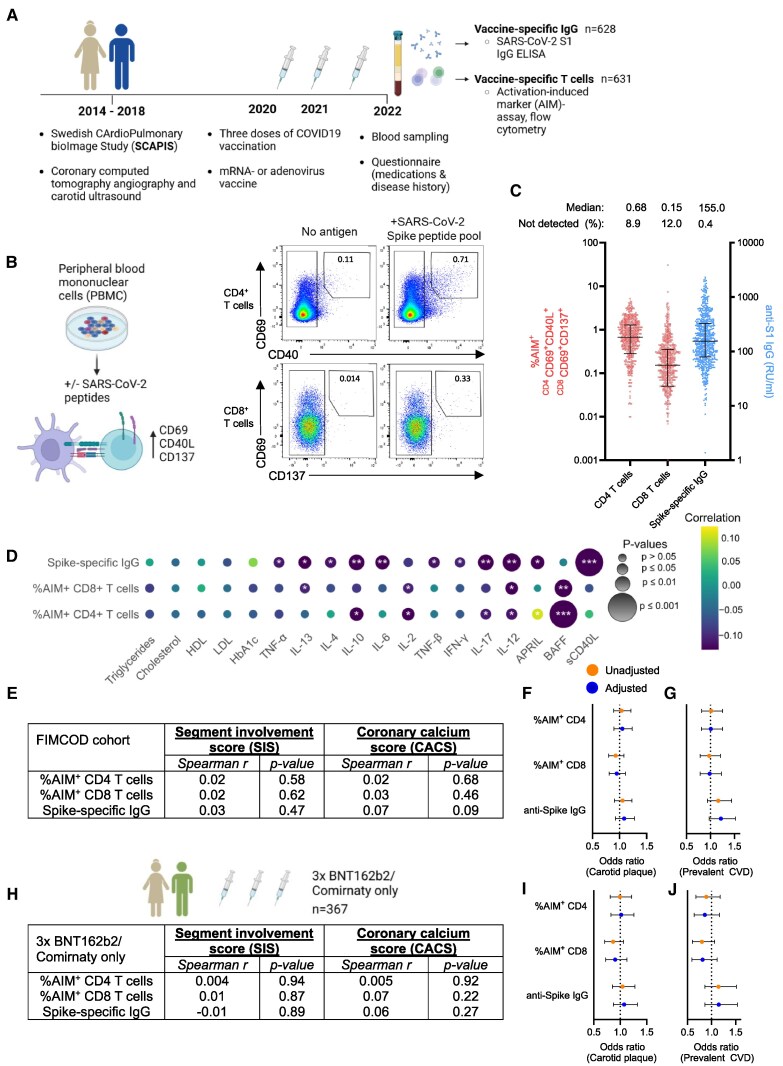
Subclinical atherosclerosis is not associated with impaired vaccine responsiveness. *(A*) Functional IMmunity and CardiOvascular Disease (FIMCOD, *n* = 644) study outline with timeline of cardiovascular imaging, SARS-CoV-2 vaccination, and blood sampling. *(B*) Outline of experiment and representative flow cytometry plot. Peripheral blood mononuclear cells (PBMCs) were cultured with or without SARS-CoV-2 spike peptide pools to detect vaccine-specific T cells. *(C*) Quantification of levels of vaccine-specific CD4 (CD69^+^CD40L^+^) and CD8 (CD69^+^CD137^+^) T cells determined by flow cytometry and plasma anti-S1 IgG measured by ELISA. *(D*) Correlation matrix of vaccine-specific immunity (AIM+ T cells and anti-Spike IgG) and clinical chemistry readouts, plasma cytokines or soluble receptors (LEGENDplex). *(E*) Associations between vaccine immunity and subclinical atherosclerosis (segment involvement score [SIS]), coronary calcium score [CACS]; spearman rank co-efficient). *(F*) Associations between vaccine immunity presence of carotid plaque (logistic regression, quartiles). Adjusted for sex, age, and time since third dose of vaccination. *(G*) Associations between vaccine immunity and history of CVD (logistic regression of quartiles). Adjusted for sex, age, and time since third dose of vaccination. *(H*) Associations between vaccine-specific immunity and subclinical atherosclerosis in study participants having received three doses of BNT162b2 (*n* = 367). *(I*) Associations between vaccine immunity presence of carotid plaque (logistic regression, quartiles) in study participants having received three doses of BNT162b2. Adjusted for sex, age, and time since third dose of vaccination. *(J*) Associations between vaccine immunity and history of CVD (logistic regression of quartiles) in study participants having received three doses of BNT162b2. Adjusted for sex, age, and time since third dose of vaccination.

We enrolled SCAPIS participants that had undergone coronary computed tomography angiography and carotid ultrasound to quantify subclinical atherosclerosis burden and had been vaccinated to take part in the FIMCOD study to assess vaccine-specific immunity and its relationship to subclinical atherosclerosis (*Figure 1* and *Figure 2A*). The average age of participants in the FIMCOD study (*n* = 644) was 68 years and they were made up of a nearly equal proportion of men and women (*Table 1*). The most common vaccine combination was three doses of Comirnaty mRNA vaccine (57%) with the combination of adenovirus-based vaccine Vaxzevria with Comirnaty or the mRNA vaccine Spikevax being the second and third most common combination. The average time between the third vaccination dose and blood sampling was 4 months (∼120 days).

**Table 1:** Baseline characteristics of the Functional IMmunity and CardiOvacular Disease (FIMCOD) study

Functional IMmunity and CardiOvacular Disease (FIMCOD) study (*n* = 644)		
Age (± SD)		68.7 (66.7–70.6)
Sex (female, %)		55
Vaccine combinations (%)	1^˚^, 2^˚^, 3^˚^ Com^a^.	57
	1^˚^, 2^˚^ Vax^b^., 3^˚^ Com^a^.	25
	1^˚^, 2^˚^ Vax^b^., 3^˚^ Spv^c^.	6
	1^˚^, 2^˚^ Com^a^., 3^˚^ Spv^c^.	5
Days since last vaccine dose (± SD)		124.0 (74.4–173.5)
LDL (mmol/L, mean ± SD)		3.5 (2.3–4.7)
HDL (mmol/L, mean ± SD)		1.6 (1.1–2.0)
Triglycerides (mmol/L, median ± IQR)		1.3 (1.0–1.7)
HbA1c (mmol/mol, median ± IQR)		37.0 (35.0–39.5)
Statins (%)		28.0
Anti-diabetic medication (%)		8.0
Anti-hypertensive medication (%)		32.6
Current smoker (%)		8.2
Segment involvement score (median ± IQR)		1 (0–3)
Coronary calcium score (median ± IQR)		1 (0–67.3)
Presence of carotid plaque (%)		69.5
History of CVD (%)		12.5
History of diabetes (%)		9.2
History of rheumatic disease (%)		4.8

^a^Comirnaty (BNT162b2, Pfizer).

^b^Vaxzevria (ChAdOx1 nCoV-19, Astra Zeneca).

^c^Spikevax (mRNA-1273, Moderna).

Percentages of SARS-CoV-2-specific AIM^+^ CD4^+^ T cells (CD69^+^CD40L^+^) and AIM^+^ CD8^+^ T cells (CD69^+^CD137^+^) were determined by flow cytometry after activation with SARS-CoV-2 peptides (*Figure 2B, C*). We did not observe any significant associations between triglycerides, lipoproteins, or HbA1c and vaccine immunity (*Figure 2D*). Instead, humoral and cellular vaccine responses were inversely associated with several cytokines (including IL-6, IL-10, IL-12, IL-13, IL-17, and BAFF) as well as soluble CD40L in plasma, indicating a potential link between low-grade inflammation and reduced vaccine responsiveness (*Figure 2D*).

Finally, we tested if subclinical atherosclerosis defined as coronary plaque burden (segment involvement score), coronary calcium score (CACS) or presence of carotid plaque was associated with vaccine-specific immunity. We did not observe any significant association between any of these measurements of subclinical atherosclerosis and the magnitude of vaccine-specific immunity (*Figure 2E, F*). Likewise, we did not observe any association between vaccine immunity and history of CVD (*Figure 2G*). Restricting our analysis to study participants receiving the most common vaccine combination (3× BNT162b2/Comirnaty, *n* = 367) did not reveal any significant associations between subclinical atherosclerosis or history of CVD and vaccine immunity (*Figure 2H–J*).

## Conclusions

Responsiveness to vaccination declines as we age and individuals above 65 years of age have diminished protection from vaccination and require more frequent boosters or high-dose vaccines.^7^ In this report, we demonstrate that inter-individual differences in immunological aging, resulting in varying abilities to respond to vaccination, does not significantly associate with pre-existing atherosclerosis or prevalent CVD. The observed association between low-grade inflammation and poor vaccine response aligns with previous reports^8^ and warrants further mechanistic studies.

There are several limitations of the present study. Vaccine responsiveness was assessed after three doses, possibly overriding immune deficits that would be visible after a first vaccine dose. The imaging of subclinical atherosclerosis was not concurrent to SARS-CoV-2 vaccination or blood sampling. Thus, we cannot exclude minor changes in coronary or carotid atherosclerosis burden between time cardiovascular bioimaging and immune phenotyping. Furthermore, we only assessed total levels of T cells responding to vaccination and did not evaluate the type of memory T cells generated or avidity of anti-Spike IgG. Additionally, our observations are limited to SARS-CoV-2-vaccines delivered during the COVID-19 pandemic.

In summary, we report that humoral or cellular SARS-CoV-2 vaccine responsiveness is not associated with subclinical or prevalent CVD burden. Further studies using other readouts of adaptive immune activation are needed to validate and expand on the generalizability of our findings.

## Data Availability

Data are available upon reasonable request.
